# Can Interoception Improve the Pragmatic Search for Biomarkers in Psychiatry?

**DOI:** 10.3389/fpsyt.2016.00121

**Published:** 2016-07-25

**Authors:** Sahib S. Khalsa, Rachel C. Lapidus

**Affiliations:** ^1^Laureate Institute for Brain Research (LIBR), Tulsa, OK, USA; ^2^Oxley College of Health Sciences, University of Tulsa, Tulsa, OK, USA; ^3^Department of Psychology, University of Tulsa, Tulsa, OK, USA

**Keywords:** interoception, heartbeat detection, panic disorder, depression, anorexia nervosa, bulimia nervosa, somatic symptom disorders, exposure therapy

## Abstract

Disrupted interoception is a prominent feature of the diagnostic classification of several psychiatric disorders. However, progress in understanding the interoceptive basis of these disorders has been incremental, and the application of interoception in clinical treatment is currently limited to panic disorder. To examine the degree to which the scientific community has recognized interoception as a construct of interest, we identified and individually screened all articles published in the English language on interoception and associated root terms in Pubmed, Psychinfo, and ISI Web of Knowledge. This search revealed that interoception is a multifaceted process that is being increasingly studied within the fields of psychiatry, psychology, neuroscience, and biomedical science. To illustrate the multifaceted nature of interoception, we provide a focused review of one of the most commonly studied interoceptive channels, the cardiovascular system, and give a detailed comparison of the most popular methods used to study cardiac interoception. We subsequently review evidence of interoceptive dysfunction in panic disorder, depression, somatic symptom disorders, anorexia nervosa, and bulimia nervosa. For each disorder, we suggest how interoceptive predictions constructed by the brain may erroneously bias individuals to express key symptoms and behaviors, and outline questions that are suitable for the development of neuroscience-based mental health interventions. We conclude that interoception represents a viable avenue for clinical and translational research in psychiatry, with a well-established conceptual framework, a neural basis, measurable biomarkers, interdisciplinary appeal, and transdiagnostic targets for understanding and improving mental health outcomes.

## Introduction

Recent interpretations of the limited impact of psychiatric neuroscience and genetics research on treatment outcomes have argued that the time has come for a pragmatic shift in focus through the identification of biomarkers with a mechanistic focus (1) and pursuit of processes that predict illness course or treatment outcome (2). In the current review, we explore how the study of interoception represents an enticing yet underutilized opportunity to apply neuroscience-based approaches to the development of novel interventions, in ways that are pragmatic and mechanistic.

We start with an overview of interoception followed by an illustration of the natural history of interoception research. We demonstrate the multifaceted nature of interoception through a focused review of the most popular methods used to study cardiac interoception. To demonstrate the links between interoception and psychopathology, we review findings in several psychiatric conditions emblematic of abnormal interoception: panic disorder, depression, somatic symptom disorders, anorexia nervosa, and bulimia nervosa. We highlight parallel findings across these disorders and areas where progress could be made. We conclude by discussing interoception’s relevance to recent paradigm shifts in biomarker research in mental health, and underscore some practical considerations relevant to the development of interoception-based mental health interventions.

## What is Interoception?

Interoception refers to the process of how the brain senses and integrates signals originating from inside the body, providing a moment by moment mapping of the body’s internal landscape. This crosstalk gives rise to urges, feelings, drives and emotional experiences under certain conditions, highlighting the importance of interoception for the maintenance of homeostatic functioning, body regulation, and survival. The enduring parcelation of the body’s processing of internal and external signals into interoception, proprioception, and exteroception first occurred more than a 100 years ago (3, 4). Since then, numerous definitions of interoception have been offered with sometimes subtle and at other times substantial variation (see Table 1). From these definitions, it is clear that interoception is a multifaceted process that can be deconstructed into different aspects such as attention, detection, discrimination, accuracy, and self report (Table 2 and Data Sheet S1 in Supplementary Material for selected definitions, and Box 1 for a discussion of taxonomy). Interoception encompasses both non-conscious and conscious levels of information processing, and the processing of painful and non-painful stimuli (Table 3).

**Table 1:** **Different definitions of interoception**.

Author(s)	Definition
Vaitl (5)	A general concept, which includes two different forms of perception: proprioception and visceroception
Cameron (6)	The afferent information that arises from anywhere and everywhere within the body – the skin and all that is underneath the skin, e.g., labyrinthine and proprioceptive functions – not just the visceral organs
Cameron (4)	Perception of the functions and physiological activities of the interior of the body
Craig (7)	The sense of the physiological condition of the body or a homeostatic afferent pathway that conveys signals from small diameter primary afferents that represent the physiological status of all tissues in the body
Khalsa et al. (8)	The perception of internal body states
Paulus et al. (9)	The central nervous system representation of visceral feelings
Couto et al. (10)	The processing of bodily signals from the viscera and somatic tissues
Critchley and Harrison (11)	Continuous dynamic feedback of afferent visceral signals that shape (the brain’s) operational functioning
Paulus (12)	A process consisting of integrating the information coming from the inside of the body in(to) the central nervous system
Barrett and Simmons (13)	The perception and integration of autonomic, hormonal, visceral and immunological homeostatic signals that collectively describe the physiological state of the body

**Table 2:** **Facets of interoception**.

Facet	Operational definition	Paradigms
Attention	Observing internal body sensations	C*, GI* Simmons et al. (14)
		R* Farb et al. (15)
Detection	Presence or absence of conscious report	C* Khalsa et al. (16)
		C* Garfinkel et al. (17)
		R* Davenport et al. (18)
		R* Paulus et al. (19)
		GI* Holzl et al. (20)
Magnitude	Intensity	C*, R* Khalsa et al. (21)
		R* Davenport et al. (18)
		GI* Herbert et al. (22)
		GI* Naliboff et al. (23)
		U* Jarrahi et al. (24)
Discrimination	Localize sensation to a specific channel or organ system, and differentiate it from other sensations	C*, R* Khalsa et al. (21)
		GI* Aziz et al. (25)
Accuracy (or sensitivity)	Correct and precise monitoring	C* Schandry et al. (26)
		C* Khalsa et al. (8)
		R* Daubenmier et al. (27)
Self-Report	Reflect upon one’s own experiences of interoceptive states, make judgments about their outcomes, and describe them through verbal or motor responses	Shields et al. (28)
		Porges (29)
		Labus et al. (30)
		Khalsa et al. (16)
		Mehling et al. (31)
		Ceunen et al. (32)
		Garfinkel et al. (33)

The illustrated paradigms cut across several organ systems including the cardiac (C*), respiratory (R*), gastrointestinal (GI*), and urinary (U*) systems (see Data Sheet S1 in Supplementary Material for detailed definitions).

Box 1 **A note on taxonomy.**

There currently exists some variability in interoception terminology. This is especially true at the self-report level, where distinctions have been made between measures of task accuracy, perceptions of task performance, and the relationship between the two (16, 32). Adding to this debate, Garfinkel et al. (33) have defined interoceptive awareness as metacognitive awareness or self-knowledge about interoceptive task performance, differentiating it from interoceptive accuracy. Others have referred to the confidence-accuracy correspondence as interoceptive coherence (34). Some have described interoceptive awareness in terms of an attentional bias (35) or simply objective task performance (36). Harmonizing these different perspectives into a unified taxonomy might provide a framework upon which comparative investigations across different psychiatric disorders could be made. We propose that the term “interoceptive insight” might reflect a more appropriate term for estimates of confidence-accuracy correspondence, particularly within the context of studying psychiatric patient populations. There are likely many additional aspects of interoceptive self-report, including questionnaires [e.g., Ref. (28, 31)] and assessments of dispositional tendencies [aka “interoceptive sensibility” (33), see Table 2]. Therefore, further parcelation of the self-report aspect of interoception represents an opportunity ripe for further investigation (37). See (37) for a historical analysis of interoception semantics.

**Table 3:** **Physiological processes that have been ascribed to interoception**.

*Non-painful*: Cardiovascular, respiratory, gastrointestinal (esophageal, gastric, intestinal, colorectal), bladder, hunger, thirst, blood/serum (pH, osmolality, glucose level), temperature, vasomotor flush, air hunger, muscle tension, shudder, itch, tickle, pleasure, genital sensation, and sensual touch
*Painful*: Visceral: kidney, pleuritic, angina, pelvic, pericardial, muscular, sickle crisis; Somatic: abscess/boil, bruising, laceration inflammation, headache (migraine, ocular, cluster); Skeletal: broken/bruised bone, and stress fracture

## The Natural History of Interoception Research

**Figure 1:**
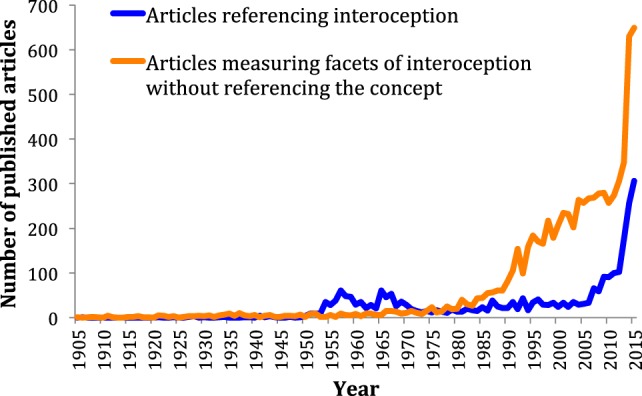
**Number of articles published on interoception versus articles published measuring interoceptive facets without directly referencing the concept**. The timeline starts in 1905, 1 year before the publication of Charles Sherrington’s book, “The integrative action of the nervous system,” which introduced the term interoception into the literature.

To examine the degree to which the scientific community has recognized interoception as a construct of interest, we identified and individually screened all articles published in the English language on interoception and all associated root terms in Pubmed, Psychinfo and ISI Web of Knowledge (Figure 1, blue line). Interest in interoception research fluctuated during most of the twentieth century followed by a stratospheric increase during the twenty-first century, suggesting this area is currently undergoing a renaissance. However, interoception is a multifaceted phenomenon that is not restricted to conscious perception or even to the human species. When we identified articles directly assessing the different facets of interoception – but without including the term interoception directly – a different pattern emerged (Figure 1, orange line, and Data Sheet S2 in Supplementary Material). It appears that interest and progress in studying interoceptive facets has been rising for the past quarter century, decades ahead of this concept, though mainly in biomedical, non-psychiatric fields. Next, we consider ways that some of these discoveries might be leveraged to advance progress in understanding the pathophysiology of psychiatric illnesses.

## Can Interoception Provide Practical Biomarkers?

Interoceptive processes have been delineated across all of the major biological systems involved in maintaining bodily homeostasis, including the cardiovascular (38, 39), pulmonary (40), gastrointestinal (41), genitourinary (42), nociceptive (43), ingestive (44), osmotic (45), thermoregulatory (46), and autonomic systems (11, 47). Many of these processes span the genetic, cellular, neural circuit, and systems levels, and they have been characterized extensively within separate branches of medicine and neuroscience. To illustrate, consider the effects of hypercapnia associated with a prolonged breath hold (as might occur when swimming underwater) or an experimental analog, inhalation of carbon dioxide: pulmonary gas exchange is altered leading to a shifting of the oxygen–hemoglobin dissociation curve, reduced blood pH, bradycardia followed eventually by tachycardia, altered body temperature, activation of medullopontine apneustic rhythm generators and limbic cortical regions eliciting the subjective experience dyspnea, negative affect, and anxiety, leading to compensatory hyperventilation and even potentially panic attacks (48–52). Accurate description of the processes at play requires holistic and integrative knowledge of respiratory physiology, basic neuroscience, functional neuroanatomy, medicine, clinical psychology and psychiatry, disciplines that are infrequently combined within one approach [see Ref. (53) for a noteworthy exception]. This example illustrates how interoception can provide a direct bridge between biological and psychological functions, one cutting across diagnostic categorizations for psychiatric disorders. It also illustrates how interoception can function as a zone of convergence between classically distinct fields. Such comprehensive approaches have been advocated previously for assessing the different components of psychiatric disorders (54) but without an emphasis on interoception *per se*. Next, we describe multifaceted approaches to interoceptive assessment.

## Multifaceted Assessment: The Example of Cardiac Interoception

To further illustrate the multifaceted nature of interoception, we focus here on one of the most commonly studied interoceptive channels: the cardiovascular system. Cardiac interoception can be defined as the process of sensing, storing, and representing information about the state of the cardiovascular system. Like all interoceptive modalities, cardiac interoception is a process that can be deconstructed into different aspects such as attention, detection threshold, symptom magnitude, accuracy, discrimination, or self-report. Each facet can be interrogated, and the literature is replete with such tasks.

**Figure 2:**
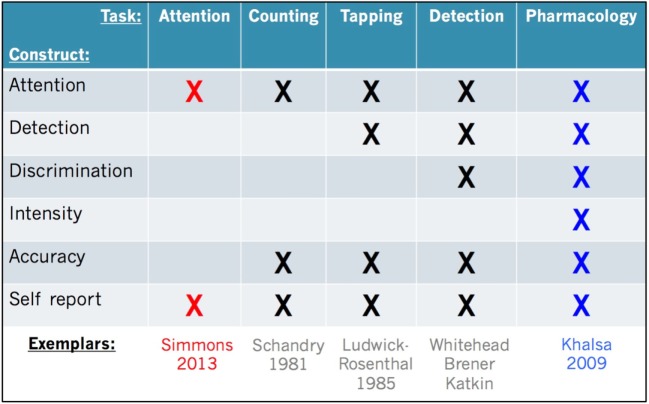
**Facets of cardiac interoception**. Representative constructs, tasks, and exemplar studies from the literature. We recommend that future studies include multiple tasks evaluating the facets of cardiac interoception from converging perspectives (e.g., red and blue highlighted tasks). An analogous approach is recommended for other interoceptive sensory modalities (see text for details).

To facilitate interpretation of the most common measures of cardiac interoception, we provide a side-by-side comparison (Figure 2). All of these tasks share in common a measurement of the cardiac signal in some form (e.g., *via* electrocardiogram or pulse oximeter). They all oblige individuals to voluntarily direct attention toward their feeling of the heartbeat (i.e., interoceptive attention), and they also require some form of self-report in order to assess subjective experience. The first tasks developed to systematically study human cardiac interoception were non-invasive. They involve silently counting heartbeats during pre-specified time periods [“heartbeat counting” (55)], or tapping the finger or pressing a button to indicate each felt heartbeat [“heartbeat tapping” (56)], or comparing the heartbeat sensation with auditory tones presented simultaneously or non-simultaneously with the heartbeat [“heartbeat detection” (57–59)]. These tasks have been characterized extensively by an outstanding generation of psychophysiologists [as summarized in Ref. (60)].

Decades of research have also revealed that each task has benefits and drawbacks. While heartbeat counting is easy to perform and analyze, the measure is potentially confounded by the influence of *a priori* knowledge of one’s own heart rate (61, 62) and a reduced sensitivity to heart rate changes (63). Heartbeat tapping provides temporal detail about each felt heartbeat (unlike heartbeat counting) but requires a more sophisticated analysis approach and nuanced interpretation and so it has not been widely utilized (64–66). Heartbeat detection is the most complex task to implement, but it yields rigorous statistical measures of accuracy at the individual subject level based on signal detection theory (16, 67–70), which has contributed to a wide popularity. A recently developed minimalistic approach involves measuring brain activity while participants simply attend to the feeling of their heartbeat sensation (14). This allows for identification of brain regions involved in interoceptive attention with less of the potentially confounding effects of somatomotor responses or interoceptive–exteroceptive stimulus comparisons (as in heartbeat detection for example). Because these tasks inherently load differently on the various facets, direct comparisons are rarely made. This may be partly related to the fact that interoception research is inherently cross-disciplinary: the same processes may be studied separately by a broad array of investigators, including psychologists, psychiatrists, physiologists, neuroscientists, and cardiologists utilizing techniques most accessible and specific to their interests and field.

One especially important point to note is that all of the aforementioned tasks typically measure cardiac interoception under conditions of physiological rest. As a consequence, in the absence of an environmental manipulation, they fail to assess one of the most important characteristics of the cardiovascular system: that it dynamically responds to perturbations in the internal and external milieu, often resulting in changes in the intensity of perceived cardiovascular sensations. In fact, when measured at rest (i.e., at a homeostatic set point), cardiac interoception is actually quite poor, with only about 35% of individuals typically able to accurately perceive their cardiac sensations (16, 60, 71–73). This low rate of detection is similar even following heart transplantation (74), which raises important questions about the neurovisceral source of heartbeat sensations. These findings suggest that under resting conditions most individuals do not actually sense their heartbeat sensations, regardless of their self-report. Since clinically and/or emotionally significant events are arousing and frequently associated with dynamic disruptions in cardiovascular homeostasis (such as palpitations during a panic attack, public speaking anxiety, traumatic hypervigilance, physical exertion, or simply excited anticipation during a roller coaster ride or horror movie), these tasks may be failing to assess an important source of symptom variability under typical measurement conditions: interoceptive intensity.

An effective method for assessing individual differences in interoceptive intensity is by modulating arousal *via* the sympathetic nervous system. Although different forms of exercise or postural changes can accomplish this (26, 73, 75, 76), such designs are limited by expectancies associated with this non-maskable manipulation. On the other hand, pharmacological manipulations have the advantage of a credible placebo manipulation that allows for measurements of responding bias and assessment during physiological resting states.

Many different compounds have been shown to alter sympathetic arousal and cardiovascular interoceptive intensity. These include isoproterenol (77, 78), sodium lactate (79–82), carbon dioxide (CO_2_) (83–87), caffeine (88–90), pentagastrin (91), doxapram (92, 93), cholecystokinin (94, 95), and yohimbine (96–98). Many of these pharmacological probes require intravenous access. They are typically applied using a chronic administration format (i.e., continuous infusion or continuous inhalation), which hinders estimates of dose–response relationships. Such studies typically rely upon concurrent or retrospective questionnaire ratings of the intensity of multiple symptoms, usually due to a focus on negative affective processes such as gauging anxiety level and/or panic attack occurrence. That is, while they might include assessments of interoceptive symptom intensity, the study focus is usually broader and many of the other interoceptive facets (e.g., detection, discrimination, accuracy) are not evaluated. Each of these probes also have different pharmacological targets, and therefore the observed sympathetic arousal changes may occur as a non-specific downstream response to physiological perturbation.

Isoproterenol is one pharmacological agent that is well suited for studying cardiac interoception. It targets the cardiovascular system *via* stimulation of beta-adrenergic receptors, and its peripheral mechanism of action provides a potent probe of afferent interoceptive processing (99). As there are beta-adrenergic receptors in the lungs, bronchodilation and associated respiratory sensations can also be elicited, though typically at a lower intensity. This allows for the assessment of respiratory interoception and illustrates the close interactions between the cardiac and respiratory systems. Some participants may report autonomic symptoms such as dizziness or flushing and/or affective symptoms such as anxiety, particularly at higher levels of stimulation, e.g., 4 μg bolus (100), or during prolonged infusion (101).

Initial psychopharmacological studies of isoproterenol were clinical in nature and investigated its anxiogenic properties by applying continuous prolonged infusions (77, 78), with subsequent studies focusing more on peripheral leukocyte sensitivity during bolus infusion administration (102–104). More recently, bolus infusions of lower doses of isoproterenol (0.1. 0.25, 0.5, 0.75, 1.0, 2.0, and 4.0 μg) have been used to study cardiac interoception in psychiatric and neurological patient samples (21, 100, 105). During this type of task, participants receive isoproterenol or saline infusions in a double-blinded protocol. They concurrently rate the intensity of perceived cardiorespiratory sensations by rotating a dial, and retrospectively rate cardiac, respiratory, and autonomic/affective symptom intensity individually. Although this task is invasive and requires physician supervision, it provides good experimental control over the cardiovascular system. It also enables accurate measurement of all facets of cardiac interoception: dichotomized dial ratings reflect interoceptive detection, cross correlations between heart rate and dial ratings reflect interoceptive accuracy, different doses provide measures of interoceptive intensity, placebo infusions enable estimates of interoceptive discrimination, and *post hoc* questionnaires assess the participant’s self-report of the task (e.g., experienced locations of perceived heartbeat sensations, task difficulty/tolerability, likelihood of drug versus placebo administration, etc.) (21). Interoceptive responses to bolus isoproterenol infusions have been well characterized in healthy populations: at high enough doses (e.g., 2 μg and above), cardiac interoceptive changes are correctly perceived by 100% of healthy individuals (8, 21, 100, 105). Neural regions supporting the perception of isoproterenol-induced cardiorespiratory sensations have also been investigated and include but are not necessarily restricted to the insular cortex, somatosensory afferent system (8, 101, 106) and the basolateral amygdala (100).

## Interoceptive Dysfunction and Prediction Errors in Psychopathology

Despite the many intersections between interoception and different fields of medicine, it is surprising that this area has received a relatively sporadic exploration of within psychiatry. Disrupted interoception is prominently featured in the diagnostic classification of several psychiatric disorders, notably anxiety and depressive disorders, eating disorders, and somatic symptom disorders (44, 107). Furthermore, interoceptive symptoms are readily accessible by patient self-report, allowing for integration in routine psychiatric assessment and treatment protocols.

New theoretical insights into how the brain perceives the body may be informative in interpreting interoceptive dysfunction in psychiatric disorders. While it is customary to believe that visceral sensations arise from direct perception of the internal state of the body *via* ascending afferent pathways, new perspectives suggest that the brain works somewhat differently. Instead of a linear translation from sensations to perceptions, it may be accurate to say that interoceptive perceptions are constructed by the brain. Interoception may actually occur through an active and iterative process of comparing the brain’s anticipation of sensation with concurrently incoming sensation, indicating that interoceptive experiences may substantially reflect predictions about the state of the body (13, 108, 109). In this view, interoceptive experience is still exemplified by ongoing visceral sensations, but it can be modified or constrained to variable degrees by the brain’s anticipatory signals. If the actual state of the body differs from the predicted state, this results in a so-called “prediction error.” When prediction errors are small or non-existent, the system can be said to be in homeostasis. When prediction errors are large, incoming interoceptive information is decoupled from the brain’s interoceptive predictions. In the setting of psychiatric illnesses like anxiety and depression this system becomes imbalanced, producing “noisy” afferent input (110). In this view, beyond simply changing the external environment, the brain may also try to reduce interoceptive prediction errors by (1) selectively attenuating the processing of incoming sensory input, (2) by triggering changes in the body that resemble the expected (i.e., predicted) input, or (3) by altering perceptual inferences about bodily states. It may be possible that interoceptive dysfunction is marked by active attempts to modulate the relative value of incoming inputs in order to restore balance, and be the basis for certain symptom reports and pathological behaviors observed in psychiatric patients such as frequent requests for reassurance, excessive treatment seeking, avoidance, or escape.

Next we consider several psychopathological conditions emblematic of abnormal interoception: panic disorder, somatic symptom disorders, depression, anorexia nervosa, and bulimia nervosa. This list is by no means inclusive, but is provided for illustrative purposes. Other relevant disorders worthy of attention that are not considered here include generalized anxiety disorder, posttraumatic stress disorder, social anxiety disorder, and others. We do not explicitly address the role of interoception in allostasis (111), stress (36), or animal and human neuroanatomy (7, 38), as these topics have been previously described in detail.

## Panic Disorder: The Prototypical Interoceptive Disorder

A survey of the literature on interoception and panic disorder suggests a somewhat conflicting picture. On one hand, most resting measures of cardiac interoceptive accuracy have failed to clearly demonstrate whether panic disorder patients perceive heartbeat sensations differently or whether they have a systematic bias toward reporting such feelings (112–114). This contrasts with many studies showing that panic disorder patients perceive cardiovascular sensations more intensely when stimulated pharmacologically *via* caffeine ingestion (88, 90), CO_2_ inhalation (115), or intravenous infusion with sodium lactate (116), yohimbine (97), cholecystokinin (117), or isoproterenol (77).

One way to reconcile these differences is in reiterating the aforementioned point that interoception under resting physiological conditions may not have as much biological salience as during non-homeostatic deviations, which can signal threats to survival – be they physical, emotional, or social. Another point is that most pharmacological studies have been conducted within the field of psychiatry, whereas most psychophysiological studies have been conducted within the field of psychology; it is therefore possible that this gap in the literature is related partly to an incomplete overlap between these disciplines. An important historical factor potentially contributing to this discrepancy is that much of the early pharmacological studies were focused on finding a single biological target, or a single pathway in the central nervous system that might explain panic anxiety. When multiple agents with different binding sites produced panic similarly, it appears that interest in this theory waned rapidly and research in this area declined. Also, the observations that panic anxiety could be effectively attenuated by medications modulating the serotonergic and/or noradrenergic systems (118–125) or GABA-ergic system (126–131), with success rates higher than most other psychiatric disorders, may have contributed to a relatively rapid shifting of funding priorities to other disorders. We should note that these developments do not rule out the possibility of identifying clinically relevant biomarkers for panic disorder. For example, respiratory muscle tension has been identified as a generator of dyspnea in individuals predisposed to panic disorder (132), and levels of cortisol have been found to moderate clinical improvement during exposure therapy for individuals with panic disorder (133).

Currently, the most common clinical application of interoceptive principles is the use of interoceptive exposure during the psychotherapeutic treatment of panic disorder (134–136). In this treatment approach, which is often implemented within cognitive behavioral therapy, the patient’s interoceptive triggers of anxiety are individually assessed in preparation for conducting interoceptive exposures. Examples include assessing dyspnea sensitivity *via* rudimentary inspiratory loading (breathing through straws of different diameters), palpitation sensitivity *via* light exercise (pushups or jumping jacks), dizziness sensitivity *via* spinning in a chair, tingling sensitivity *via* hyperventilation, and flushing sensitivity *via* sitting in front of a heater. Anxiety levels during each manipulation are recorded, generating a rudimentary “interoceptive profile.” During the ensuing exposure phase patients voluntarily choose to experience these sensations in a repeated fashion, gradually working their way up a hierarchy to their most anxiogenic triggers in a process intended to elicit habituation and extinction to interoceptively triggered panic, resulting in reduced maladaptive avoidance and escape behaviors. In the outpatient clinic setting, these manipulations are variably effective at eliciting anxiety, with some patients being more sensitive to respiratory cues and others more sensitive to cardiac or dizziness. Some patients develop rapid tolerance to these measures despite the presence of continued panic attacks.

Despite the established clinical efficacy of standard interoceptive exposure for anxiety disorders, it is noteworthy that this technique suffers from limited utilization with only 12–20% of psychotherapists reporting including it in their treatment approach (136). This has led some to refer to interoceptive exposure as “the single least utilized evidence-based anxiety treatment” (137). Putative barriers to utilization include a lack of training sites, logistical hurdles (e.g., occasional need for exposure durations longer than a standard therapy session), policies against conducting exposures outside of the workplace setting, and perhaps most tellingly, negative therapist beliefs (e.g., that interoceptive exposures are unethical, intolerable, or even harmful) (137).

The clinical evidence reviewed here implies that individuals with panic disorder may have differential sensitivity to interoceptive cues producing increased arousal. It also begs the question of whether utilizing more potent cues during interoceptive exposure could boost the efficacy of this intervention. For example, panic disorder patients show increased autonomic arousal and heightened anxiety during exposure to experimental paradigms involving non-pharmacological approaches (being trapped in a small dark chamber) (138) as well as pharmacological ones (oral caffeine ingestion) (139). There is experimental evidence that pharmacological interoceptive exposure therapy can reduce anxiety disorder symptom severity either as a monotherapy (84, 140–142) or as an augmentative approach (143). Finally, there is the question of whether modulating both physiological homeostasis *and* the perception of controllability might further improve the ecological validity and efficacy of interoceptive exposures (144). However, there are few studies to date, the impact of such interventions on longer term outcomes (e.g., 6 months or beyond) are unknown, and none of these approaches have translated into regular clinical practice.

Substantial barriers to implementation of an augmented approach exist under standard clinical conditions. For example, approaches using more potent interoceptive exposures, such as CO_2_ inhalation or isoproterenol infusion, carry with them increased requirements for medical screening and monitoring, reduced applicability in patients with significant medical comorbidity, and undoubtedly would be more expensive. The evidence base to date is insufficient to determine whether augmented approaches might be worth considering in a standard clinical setting. This might change if studies showed a substantially superior efficacy to standard interoceptive exposures. Despite these challenges, interoceptive exposure is currently a well established tool for the treatment of panic disorder. It has been integrated into transdiagnostic treatments, and it is being increasingly emphasized as an effective strategy (136).

Finally, there are currently no interoceptive biomarkers in clinical use to monitor outcomes and track illness course in panic disorder. This raises several questions. With all of the history of work in this area, why has not there been further progress? Can interoceptive biomarkers truly improve the characterization or treatment of panic disorder? With all of the focus on the physiological mechanisms of interoception, can we afford to ignore the fact that psychological processes can also provide considerable therapeutic contributions? (144–148) These questions highlight some of the opportunities and challenges for translational development of exposure-based treatments for panic disorder.

## Dampened Interoceptive Experience in Depression

Interoceptive dysfunction has been theoretically linked to depression and major depressive disorder (MDD) [for reviews, see Ref. (35, 110)]. In aggregate, the available data seems to suggest that depression is associated with blunted cardiac interoceptive awareness. Women with MDD show lower accuracy on heartbeat counting (149). MDD patients of both genders display lower accuracy than healthy comparisons on a heartbeat counting task (150). Performance on heartbeat counting tasks has been negatively correlated with symptoms of depression (151), and in a separate study, associated with reduced positivity and reduced decision-making ability (149). Depressed patients also exhibit comparatively lower heartbeat counting accuracy than patients with panic or anxiety disorders (152).

Poorer resting heartbeat interoception is not always seen in depression. In a study comparing healthy participants to moderately depressed and severely depressed patient samples, the severely depressed sample showed higher heartbeat counting scores than the moderately depressed sample (153). Although the authors controlled for many aspects of individual differences between the moderately depressed community and clinical population, as a group, the more severely depressed patients were also higher in anxiety, which might have explained differences in accuracy. Indeed, in a follow-up study by the same group, poor heartbeat counting accuracy was observed in a depressed sample but found to increase as a function of reported anxiety (154).

Interpreting the varying heartbeat counting findings in depression requires some conjecture. Given that most individuals are unable to feel their heartbeat sensations at rest, one possibility is that scores on this task are prone to a certain form of exaggerated symptom report. One can speculate that in the setting of anxiety, symptom reports are biased by the issuing of increased or exaggerated anticipatory prediction errors, which produces higher counts and higher accuracy scores. In depression on the other hand, interoceptive prediction errors might operate in the opposing direction, leading to lower counting rates.

In both cases, the insular and anterior cingulate cortices would be considered key structures involved in generating prediction errors, and indeed there is evidence of altered functioning in these regions in depression (155, 156). In a noteworthy study, Avery et al. (157) found that depressed patients displayed decreased activation in the dorsal mid-insula when attending to interoceptive sensations relative to healthy comparisons, and connectivity between the dorsal mid-insula and other limbic structures predicted depression severity. In a different study using heartbeat counting, depressed patients showed reduced activity in the anterior insula, and this signal correlated with depression severity (158). Interpreting these findings within an interoceptive prediction error framework, it appears possible that the interoceptive attention only condition [i.e., Ref. (157)] may have activated a more posterior sensory region of the insula (in fact, directly at the termination site of vagal afferent projections) whereas the heartbeat counting condition, which can be more sensitive to bias (61, 62), activated a more anterior region of the insula. One can speculate that the anterior insula signal directly reflects the source of interoceptive predictions, and therefore could represent a key contributor to prediction error signals. A way to verify this would be to perform both the interoceptive attention and heartbeat counting tasks in the same individuals and determine whether the same anterior–posterior gradient of activation was observed. Another way would be to study neural processing during the anticipation and receipt of a homeostatic perturbation, and evaluate whether differential anterior insular activation during the anticipatory but not perturbation period occurred for one patient group versus another.

With respect to homeostatic perturbations, studies in depressed patients are limited, and findings are mixed. Some studies suggest hyperactive sympathetic responding and others suggesting attenuated responsiveness [see Nemeroff and Goldschmidt-Clermont (159) for a review]. In response to an oral yohimbine challenge patients with MDD reported higher levels of palpitations, restlessness, flushing, sweating, tremors, and anxiety than healthy comparisons (160). In a different study, patients with depression were more tolerant than panic disorder patients to the effects of inhalation of 5% and a 7% carbon dioxide despite exhibiting similar increases in respiratory rate and tidal volume (85). This latter finding provides additional evidence of dampened interoceptive reactivity in MDD compared to panic disorder, though the rationale for why this was observed for a respiratory as opposed to cardiac challenge is unclear.

These findings have contributed to several theoretical models of depression in which altered interoception is a putatively key phenomenon. Paulus and Stein (110) characterize this as an altered signal to noise ratio with noisy interoceptive input at baseline preventing individuals from gaining an accurate picture of their internal bodily landscape. Northoff et al. hypothesize that decreased sensitivity to exteroceptive stimuli results in reduced neural activation to exteroceptive signals, leads to an imbalance between the processing of interoceptive versus exteroceptive signals, and a relative increase in the prominence of interoceptive information (161). Harshaw (35) attempts an integration of these views, by discussing three potential interoceptive “pathways” to depression: direct modification of insula functioning, decreased sensitivity to social cues causing interoceptive changes, and disruptions in interoceptive attentional capacity perhaps related to increased rumination. While further studies are needed to ascertain the veracity of these theoretical models, a recent finding that the insula and putative reward regions show differential responses based on whether appetite has decreased or increased in depression (poorer response the former and increased response in the latter) suggests a more complicated picture with the potential for multiple subtypes based on neural and behavioral symptom clustering (162).

Finally, altered immune function has also been observed in depression (163, 164). While immune processing clearly represents an important form of interoceptive processing, a detailed discussion is beyond the scope of the current review. It is worth simply noting that from a methodological standpoint, infusions of inflammatory markers (e.g., endotoxin) have been observed to stimulate rapid but transient changes in mood (depressed mood), increased autonomic tone (temperature and blood pressure), and cytokine production (165). This type of approach may represent another viable experimental framework for investigating relationships between depression, immune function, and interoception.

## Heightened Interoceptive Signaling in Somatic Symptom Disorders

Somatic symptom disorders have received less attention in the interoception literature compared with other areas of psychopathology. Reviewing this literature is somewhat complicated by the recent change in categorization from somatoform disorders in DSM 4 to somatic symptom disorder and related disorders in DSM 5 (166). The fact that somatization is quite common in anxiety and depressive disorders poses an additional challenge to disentangling symptom reporting due to somatic symptom disorder versus those stemming from affective processes (167, 168). As a result of these considerations, we have widened our discussion to include pain processing among patient and non-patient populations, as well as those showing evidence of altered somatosensory responding.

Despite the exaggerated symptom reports, interoceptive accuracy seems to be poor in somatic symptom disorders. Patients with psychosomatic disorders score lower on heartbeat counting tasks than healthy controls (169). Medical outpatients being evaluated for palpitations also exhibit low resting heartbeat detection accuracy despite a tendency to overstate their experience of palpitations (170). They also have high psychiatric comorbidity: in a small study, over 25% of these had a lifetime prevalence of panic disorder, and almost 50% had a lifetime prevalence of another psychiatric disorder (171). Medical outpatients who were more anxious and stressed reported physical symptom discomfort at lower heart rates during an exercise treadmill test, indicating poorer interoceptive distress tolerance (74). These elevated comorbidity rates and poorer interoceptive distress tolerance raise the possibility that both panic and somatic symptom disorders share some overlapping dysfunction, perhaps in neural circuitry underlying the heightened attention toward and negative interpretation of interoceptive signals.

Environmental context also appears to play a role in symptom reporting in somatization. For example, during a rebreathing test intended to increase pCO_2_ levels in the blood, high symptom reporters performed accurately when given neutral cues about the stimulus (“the gas mixture might alter breathing behavior and induce respiratory *sensations*”) but when given negative harm cues about the stimulus (“the gas mixture might alter breathing behavior and induce respiratory *symptoms*”) they performed worse and endorsed higher dyspnea (172). Another study showed that individuals reporting high amounts of physical symptoms exhibited a bias toward overestimating the intensity of inspiratory breathing loads when they were closely spaced together (173). It seems plausible that interoceptive prediction error signaling might explain some of these exaggerated symptom reports, such that the symptom report is generated by an inappropriately heightened sense of anticipation.

Interoception has also been studied in relation to somatosensory amplification, or the propensity to interpret signals arising from within the body as disproportionally intense, noxious, or disturbing (174). Patients with non-cardiac chest pain have higher somatosensory amplification scores despite similar heartbeat detection accuracy compared to a sample of patients with cardiac chest pain (175). They also have higher healthcare utilization and poorer outcomes (176). In a cross sectional study of medical outpatients, somatosensory amplification was positively associated with self-reported body awareness, somatic symptoms, and trait anxiety (177). Despite these findings in clinical populations, healthy participants performing better than chance on heartbeat detection had significantly lower scores on the somatosensory amplification scale (178, 179).

Somatosensory amplification involves more than interoception. It includes heightened self-scrutiny, increased attention to weak or infrequent bodily sensations, the tendency to appraise ambiguous stimuli negatively, and to interpret them as pathological or symptomatic of disease (180). Since several of these processes, including negative self appraisal and heightened self scrutiny, are also features of anxiety and depression (181), it seems possible that the tendency to over endorse interoceptive symptoms in somatic symptom disorders could be driven by overlap between the conditions. On a more parsimonious note, symptom reporting in both types of disorders may be driven by a similar source – increased anticipatory neural signals in interoceptive regions of the brain including the insula and anterior cingulate. Once can speculate that reduced interoceptive accuracy in the presence of exaggerated symptom reporting might reflect the presence of interoceptive prediction errors in these illnesses, and an associated pathology in the brain’s predictive machinery. This could account for how exaggerated bodily symptom reports generate the prototypical behavior of these disorders: repeated treatment seeking for a medical explanation. This view also offers a novel framework from which to extend our understanding of the neural basis of these “functional” disorders by applying the methodology of prediction.

Identifying the presence of interoceptive prediction errors in somatic symptom disorder patients might also provide a basis for treatment. A review of interoceptive fear conditioning in chronic pain conditions suggested that the fear of pain stemming from interoceptive prediction errors may be a powerful motivator in panic disorder *and* somatic symptom disorders (182). Furthermore, interoceptive fear was found to mediate the relationship between body vigilance and chest pain for non-cardiac chest pain patients (183). Whether interoceptive exposure therapy could extinguish fearful apprehensions of internal bodily sensations in somatic symptom disorders is presently uncertain. However, one expectation following from such a finding would be that somatic symptom exaggeration would be markedly attenuated following successful treatment in these patient populations, and would be associated with a reduced frequency of inappropriate treatment seeking. Exposures could even be tailored to a patient’s individual symptoms. For example, if a patient’s concerns were centered around non-cardiac chest pain, exposures pertaining to cardiac events could be designed (running on a treadmill, pharmacological manipulation). Assessment for symptom attenuation and/or reduction in frequency of unnecessary medical presentations would be conducted routinely to document and evaluate progress. If the interoceptive exposures did in fact reduce symptom reporting, not only would this novel approach be useful in treatment, but it would provide further support for an etiological link between disrupted interoceptive awareness and somatic symptom disorders.

## Biased Interoceptive Signaling in Anorexia Nervosa

Ever since the term anorexia *nervosa* (AN) was coined (184), clinical descriptions of AN portrayed interoceptive disturbances: patients with AN were apprehensive not just about eating food but also about their bodies. And while they were preoccupied with abnormal perceptions of their body exterior, as in the “fear of becoming fat,” they also frequently worried about abnormal experiences happening inside of the body. Classic case reports even emphasized “vague feelings of fullness” (185) or “a failure of recognizing bodily states” (186) as key illness characteristics. This has led to prominent modern theoretical accounts supporting the idea that interoception in abnormal in AN (44, 187–189).

Interoceptive dysfunction in AN can also be located within descriptions of “emotion dysregulation” (190), “poor distress tolerance,” (191) and perhaps most tellingly, “alexithymia” or the inability to discriminate between emotional states and bodily sensations (192, 193). Despite these perspectives, the phenotype of external body image disturbance continues to permeate diagnostic nosology. It remains a core feature of AN in DSM 5, leaving the phenotype of interoceptive dysfunction in AN comparatively unexplored.

At the self-report level, the Eating Disorder Inventory (EDI) (194) is a tool intended to assess psychological and behavioral traits in AN and bulimia nervosa [for a review, see Ref. (187) as well as (195–200)]. It has eight subscales, one of which was originally called the “Interoceptive Awareness” (IA) subscale. Abnormal scores on this subscale have been associated with AN for the original and subsequent versions, when it was renamed the “Interoceptive Deficits” subscale (201), and its popularity is reflected by translation into multiple languages (202). A large prospective study even found that low scores on this subscale predicted risk for developing an eating disorder among 664 adolescent girls (203). In fact, scores on this subscale show the best sensitivity and specificity of all the subscales at discriminating individuals with AN from healthy comparisons (202).

Despite the popularity of the EDI measure, there are a number of challenges posed by interpreting the IA subscale as a valid measure of interoception in AN. (1) It comprises 10 questions, only 2 of which directly refer to physiological states: confusion about hunger, and bloating after a small meal. The remaining questions assess difficulty in recognizing and labeling feeling states, a process more akin to alexithymia (204–206). Since the IA subscale also correlates significantly with alexithymia in AN (205), equating differences in self-report on this subscale to differences in visceral perception is challenging. (2) Variability in interoceptive subscale scores has also been observed. For example, retrospective studies of recovered AN individuals have reported abnormally lower (207) but also abnormally higher scores (195, 199) relative to healthy comparisons. Interpreting these differences represents a methodological challenge. (3) Several biological parameters have also been associated scores on the IA subscale (196–198, 200, 208), but these parameters do not conceptually fit together easily, limiting the clinical interpretability of these observations.

Beyond interoceptive self-report, few studies have examined if cardiorespiratory interoception is impaired in AN. Patients with AN have been found to be less accurate at counting their resting heartbeat compared to an age- and sex-matched comparison group (209), whereas short-term food deprivation in healthy females increased heartbeat counting accuracy (210). In a larger but mixed sample of eating disorder patients using a different task, heartbeat detection, there was no evidence for differences in resting interoceptive accuracy (211). Methodological differences aside, none of these eating disorder studies assessed interoception across the range of physiological and affective perturbations relevant to homeostasis, meal consumption, and emotional experience.

In our initial study of interoceptive processing in AN, we applied randomized, double-blinded infusions of isoproterenol and saline during a pre-meal anticipatory state (expectation of eating a 1000 Calorie meal) and immediately after consuming said meal (21). We found that individuals with AN experienced cardiorespiratory sensations abnormally intensely, especially the sensation of dyspnea, but only during the meal anticipation period. The meal anticipation period was also marked by exaggerated detection of interoceptive symptoms during the saline infusions, i.e., false positives, when no cardiorespiratory changes had occurred. This provided the first promising evidence supporting the idea that interoceptive prediction signaling is distorted in AN, as previously suggested by Kaye et al. (44). Abnormal interoceptive detection under conditions of expectation provides evidence supporting the notion that AN is a condition where, similarly to anxiety and depression, the baseline is “noisy” and signals are obscured (110). However, the findings from this study also suggest that this noisy baseline appears to be a contextually driven state: one modulated in part by the interaction of environmental, disorder-specific cues (anticipation of meal consumption), with changes in adrenergic tone. Whether altered interoceptive prediction signaling during meal anticipation contributes to phenotypes of high anxiety in AN or alternatively, might be explained by enhanced meal associated anxiety, is presently unknown.

Evidence for aberrant interoceptive encoding at neural levels in AN can be found in neuroimaging studies. Aversive interoceptive provocation consistently identifies a network involving the insula, anterior cingulate, somatosensory cortices, and amygdala in healthy individuals (101, 117, 212, 213). This putative “aversive anticipatory neurocircuit” shows abnormalities in eating disorders. For instance, individuals who have recovered from AN (i.e., achieved a normal weight) demonstrate increased activation of the insula and anterior cingulate when anticipating anxiogenic stimuli – viewing food images (214, 215) or painful esophageal stimulation (216), but perplexingly, exhibit *decreased* insular cortex activation relative to healthy controls when actually tasting a food stimulus (217). This exaggerated anticipatory neural response but dampened response to body stimulation in AN has been explained as a deficit in the integration of expected versus experienced bodily experiences – i.e., as an interoceptive prediction error (44, 218, 219).

It is also worth noting at this point that the prevalence of anxiety in AN has been increasingly underscored in epidemiological and genetic studies. Anxiety disorders are well known antecedents to the diagnosis of AN (220, 221). They have a nearly 80% lifetime comorbidity with AN (222), with generalized anxiety disorder showing the highest point prevalence occurring in 40% of patients with AN (223, 224). Anxiety disorders even occur disproportionately in first-degree relatives of individuals with AN (225, 226). Collectively this has led to the suggestion that anxiogenesis contributes substantially to the risk of developing eating disorders (227, 228).

As noted previously, interoceptive prediction errors have also been hypothesized to explain core features of anxiety disorders (110, 218), perhaps because the anticipation of aversive stimuli activates the same regions: insula, anterior cingulate, amygdala, and somatosensory cortex (229, 230). These regions show *increased* activation in anxiety disorders during anxiety evocation (231–234) and also during anxious rumination in AN (235), suggestive of shared dysfunction. Despite these and other indications of links between anxiety and eating disorders (236), there is a distinct lack of research into avenues of potentially shared pathophysiology, and the degree to which abnormalities in anticipatory neurocircuity overlaps across these conditions is unknown.

In terms of clinical implications, exaggerated interoceptive bias during pre-meal states in AN raises the question of whether interoceptive sensations might represent potent intermediary physical symptoms worth highlighting and targeting in treatment, particularly surrounding feeding times. For example, meals are an inherently anxiety provoking experience in this population that may reinforce their ability to avoid eating. In this regard, it seems plausible that integrating interoceptive exposures into treatment for AN might be effective for extinction learning approaches employing food to extinguish conditioned responses (227, 237). Current treatments grounded in principles of desensitization and extinction learning through repeated exposure to feared food cues (238, 239) have shown modest benefits on reducing pre meal anxiety and improving meal consumption (237, 240, 241). We propose that it might be possible to develop novel food-focused fear extinction procedures by pairing such treatments with pharmacologically augmented exposure to interoceptive sensations. For example, the repeated application of isoproterenol infusions during the pre meal period might reduce biased interoceptive anticipatory responding (BIAS) in AN by reversing fearful stimulus–response associations with eating. If reducing BIAS makes the experience of eating less fearful and more tolerable for individuals with AN, it might help to reduce the intense fear of gaining weight that is a core feature of this illness. Such a discovery would pave the way for clinical trials investigating the potential impact of reducing BIAS on improving weight restoration and maintenance, representing a novel approach forward in the treatment of this devastating illness.

## Distorted Interoception in Bulimia Nervosa

Disturbed interoception has been consistently considered to be a feature of bulimia nervosa (BN). Many of these studies have been based on self-report measures and diagnostic interviews comparing individuals with BN to healthy comparisons. For example, at the self-report level patients with BN endorsed more items on the eating disorders inventory (EDI) interoceptive awareness subscale than healthy controls (242). Furthermore, patients with BN exhibit associations between anticipatory anxiety, interoceptive awareness (assessed *via* the EDI), and body image distortion, a common feature of eating disorders (236).

In response to sodium lactate and isoproterenol infusions, individuals with bulimia nervosa report higher panic and anxiety symptoms than healthy control subjects, despite experiencing a similar increase in sympathetic response (243). This would seem to align with the self-report observations above, and also suggests heightened interoceptive awareness and a negative attribution style toward interoceptive sensations. However, under resting physiological conditions, recovered BN patients have been observed to have reduced heartbeat counting accuracy (244) [but see Pollatos and Georgiou (245)]. Reduced sensitivity to other types of internal and external sensations have also been documented in BN. Patients with BN demonstrated increased thresholds for heat pain, particularly those who were engaged in binging and purging (246). Gastric capacity was also found to be larger in BN relative to obese patients or healthy comparisons (247). Altered gastric interoception was further supported by evidence for differential responding to satiety signaling in a consumption paradigm: patients consumed significantly more of a yogurt shake than healthy comparisons, yet reported similar satiety ratings (248).

Neuroimaging studies also provide some support for altered interoceptive function in BN. Patients with BN showed increased activity in the insula and anterior cingulate cortex in response to food viewing relative to healthy comparisons (249). Even after recovery, women formerly affected by BN had high activity in the right anterior insula in response to sweet tastes, as compared to healthy comparison women (217). Compared to controls, patients with BN showed increased gray matter volume in the left orbitofrontal gyrus and the left anterior ventral insula (250), regions that have been implicated in taste processing and interoception, respectively. Beyond the insula, somatosensory regions have also been implicated in the experience of interoceptive awareness (8, 101). Patients with BN have shown evidence of increased volume in somatosensory regions relative to healthy comparisons (251).

Although the aforementioned studies provide some initial evidence of dysfunctional interoceptive representation in BN, the pathophysiology underlying these patterns is far from clear. It might seem from the self-report and neuroimaging literature that there is a pattern of hypersensitivity to interoceptive signals in BN. However, the peripheral physiological evidence indicates a tendency toward both blunted and exaggerated responses to interoceptive perturbation. These contradictory findings also fail to disentangle potentially differing neural circuits underlying binging (and associated urges) and purging (and associated emotional states such as guilt) (252).

There are also likely to be heterogeneous autonomic effects related to repetitive binging and purging. One can speculate that the large shifts in autonomic tone during repeated binging and purging might cause conditioning effects – habituation or even sensitization – toward internal bodily states. For example, it has been demonstrated that one unconditioned response, salivation due to food presentation, is attenuated in BN and normalizes following treatment (253). As an example of potential peripheral sensitization, individuals with BN show decreased baseline sympathetic tone yet increased adrenergic sensitivity to isoproterenol (254). Prospective longitudinal studies of multilevel interoceptive processing in BN individuals across illness and recovery states might help to clarify the underlying pathophysiological transformations.

With respect to treatment, interoceptive exposure therapy in BN has primarily focused on repeated exposure to binge or purge cues (e.g., consuming a “forbidden” food or a large quantity of food without being allowed to purge). While this approach has shown increased efficacy over relaxation treatments, as many as 50% of patients remain ill at long-term follow up (255, 256). In this context, it is worth noting that patients with BN tend to show the highest incidence of panic disorder comorbidity among eating disorders (224). Whether augmented interoceptive exposure treatment to reduce anxiety sensitivity would boost the efficacy of this form of treatment in BN is unclear. Next, we discuss the relevance of interoception to recent paradigm shifts in biomarker research in mental health.

## Interoception and RDOC

The implementation of the Research Domain Criteria (RDoC) by the NIMH can be viewed as an attempt at the organizational level to improve the discovery of novel biomarkers of psychiatric illness (257). Rather than focusing on clinical observations as a basis for diagnosis and selection of research samples, RDoC relies on targeted investigations across multiple dimensions of different biological systems to understand their relative contributions to psychiatric pathophysiology. Interoception is inherently compatible with this framework, as it provides a direct bridge between biological and psychological processes in numerous mental health conditions. It appears to intersect with multiple RDoC domains, especially the arousal/regulatory systems, negative valence systems, and cognitive systems.

Given the broad systems covered by interoception, it could even be argued that interoception should represent its own domain. For instance, assessments of cardiorespiratory interoception can be readily mapped into the multiple RDoC levels of Self-report [e.g., Multidimensional Assessment of Interoceptive Awareness scale (31)], Behavior (e.g., heartbeat intensity rating), Physiology (e.g., heart rate, breathing rate, and blood pressure response), Neural Circuits (e.g., insula response) (8, 14, 101), Cells (e.g., glomus cells in the carotid sinus, baroreceptors in the aortic arch) (47), Molecules (e.g., beta-adrenergic receptor agonism), and Genetics [e.g., Single Nucleotide Polymorphisms associated with autonomic hypersensitivity (258)]. An occasionally overlooked point is that the RDoC framework is not set in stone. At its inception a number of domains were proposed, and associated constructs and sub-constructs were defined during workshop proceedings according to workgroup consensus. While the resulting matrix covers many areas of research in psychiatry, some key gaps remain and at the organizational level NIMH has expressed openness to adding domains in the future. In the following section, we consider practical issues pertaining to the assessment of interoceptive constructs.

## Existing Tests with Known Construct Validity

The interoceptive tests with the greatest construct validity appear to be those in which a homeostatic perturbation is induced and compared with the associated physiological change and symptom report. Many of these are somewhat invasive due to the inherent nature of studying an internal bodily process, and they can sometimes induce negative affect. Examples on the more invasive spectrum include anorectal balloon distension to assess gastrointestinal sensitivity (23), urinary catheterization to assess bladder sensitivity (24), and intravenous infusions of isoproterenol to assess cardiac and respiratory sensitivity (16). Less invasive examples include carbon dioxide inhalation (83–87), transient inspiratory breathing restriction (18, 19), and the cold pressor task (259). These latter tasks are well suited to studying interoception in unique populations that might not tolerate a more invasive approach, such as children and adolescents (86, 260) or those with severe medical comorbidity. Tasks on the least invasive spectrum are the most suited to widespread use across diverse participants, but they tend to suffer from floor effects, something especially true with cardiac interoception (16). One noteworthy exception may be the combination of interoceptive attention tasks with functional neuroimaging, which have demonstrated insula activation in health samples (14, 15) as well as psychiatric patient samples (157, 162, 235).

All of the aforementioned approaches have good psychometric properties due to the ability to produce parametric perturbations in either dose, load, or inflation pressure. In other words, as the levels of stimulation increase, subjective reports of domain specific intensity reliably and reproducibly increase. However, despite good construct validity, there is limited data available regarding test–retest reliability. Additional studies are also needed to evaluate individual differences in cross modal interoception [e.g., Holzl et al. (261); Herbert et al. (22)]. The following represents a representative review illustrating some of the best available data on interoception reliability within specific interoceptive facets.

A study of heartbeat detection involving two visits across separate days observed only a moderate correlation between accuracy across visits (*r* = 0.45, *p* < 0.0003, Cronbach’s alpha = 0.62) (262). In that study, age accounted for 30% of the variance in interoceptive accuracy. A study of the relationship between interoceptive accuracy on heartbeat counting versus heartbeat detection in the same subjects found a low correlation between the tasks (*r* = 0.316, *p* = 0.004) (33). An isoproterenol study of heartbeat perception, involving two bolus infusion sessions in the same day separated by a meal, found that accuracy scores were significantly correlated across the different meal points in both the anorexia nervosa and healthy comparison groups (21). For example, both groups displayed moderate positive correlations between pre and post meal measurements at the 2 mcg dose (HC: *r* = 0.58, *p* = 0.02; AN: *r* = 0.47, *p* = 0.009) and showed high correlations at the 4 mcg dose (HC: *r* = 0.72, *p* = 0.003; AN: *r* = 0.86, *p* < 0.001). A study of bladder interoception using two scales to measure bladder fullness versus urination urge found that ratings on each scale were significantly related to bladder volume, with good to excellent intraclass correlations across two 3-day periods (ICCs typically 0.7 or greater). However, in that study, the setting in which ratings were obtained substantially impacted the agreement between subjective ratings and observed voided volume. Patients with overactive bladders and healthy volunteers both tended to rate bladder fullness and urinary urge higher when they were observed in a hospital setting than when measurements were taken at home. A study using the Multidimensional Assessment of Interoceptive Awareness (MAIA) scale as a self-report measure of interoception in chronic low back pain found that Cronbach’s alphas for six of the eight MAIA scales ranged from 0.74 to 0.90 across the total sample (263). In the next section, we highlight important criteria to consider when selecting measures of interoception.

## Important Selection Criteria for Interoception Tasks

It is highly advantageous to select measures that allow for perturbation of the interoceptive system being targeted. This allows for parametric modulation and simultaneous comparison with symptom report, physiology, and nervous system function. However, since such paradigms are often intricate, it is important to also measure symptoms in a minimalistic manner to allow for naturalistic assessments of symptom report, physiology, and nervous system function. We recommend a combination of the two approaches. For example, if targeting cardiac interoception in anorexia nervosa, it might be optimal to measure cardiac sensitivity using both an interoceptive attention task [as in Kerr et al. (235)] and separately, an isoproterenol infusions task [as in Khalsa et al. (21)]. If targeting respiratory interoception, it might be optimal to measure respiratory sensitivity using both an inspiratory breathing load task [as in Paulus et al. (19)], and a carbon dioxide inhalation task [as in Pine et al. (86)].

Using tasks which dynamically perturb interoceptive systems have four major advantages: (1) they allow for the identification of interoceptive phenomenon not present during baseline states (4). (2) They bypass the limitations of floor effects, which are especially well documented for the cardiovascular system. (3) They facilitate the manipulation of environmental context and expectancies in a mechanistic fashion. This approach is ideal for investigating the impact of perceived controllability on symptom reports (144). (4) They are more naturalistic. Interoceptive exposure manipulations in the outpatient clinic setting are often somewhat constructed (e.g., breathing through straws of different diameters, doing pushups or jumping jacks, spinning in a chair, sitting in front of a heater). They are also sometimes variably effective at eliciting anxiety: some patients may show greater sensitivity to respiratory signals and others more to cardiac signals or dizziness. Some patients may also develop rapid tolerance to these measures despite the continued presence of elevated anxiety and/or panic attacks (the presence of residual or surreptitious avoidance behaviors must first be thoroughly excluded). In such cases, interoceptive modulations might potentially boost the efficacy of exposure therapy. For example, there is experimental evidence that pharmacological interoceptive exposure therapy can reduce anxiety disorder symptom severity either as a monotherapy (84, 140–142) or as an augmentative approach (143). However, with few studies to date, the impact of such interventions on longer term outcomes (e.g., 6 months or beyond) are unknown, and none of these approaches have translated into regular clinical practice. Developing augmented interoceptive exposures might therefore potentially boost the efficacy of interoceptive exposures by using more potent stimuli that modulate both physiological homeostasis *and* the perception of controllability.

We would also recommend collecting multiple organ system measures of interoceptive modulation, in order to develop an “interoceptive profile” of a person. This could be accomplished by independently modulating the cardiovascular, respiratory, gastrointestinal, urinary systems, and comparing the self-report data with simultaneous measures of behavior, physiology, and neural circuits, all within the same individual. Conducting such structured and systematic investigations could yield substantial insights into the pathophysiology underlying psychiatric disorders marked by dirsturbed bodily processing.

## Minding Practicality in Interoception Research

Ultimately, for interoception measures to become meaningful, they must allow researchers to test strong hypotheses about clinically relevant issues. One practical example of future interoceptive exposure-based therapy might be to assess a patient’s responses to interoceptive challenges across several systems (e.g., cardiovascular, respiratory, and gastrointestinal) to obtain real time calibrated dose–response measures of physiological dysfunction – not simply negative affect induced by a stimulus. This could provide a patient-specific “interoceptive profile” delineating where abnormalities occur: perhaps a noisy baseline (increased false positives during placebo stimulation) or hypersensitivity (left-shifted dose response curve). Subsequent treatments could involve interventions designed to normalize interoceptive and affective imbalances, with efficacy acutely assessed *via* profile normalization (attenuating baseline variability or shifting the interoceptive dose response curve to the right) and chronic efficacy maintained *via* tolerance of periodically repeated challenges. To be clinically meaningful, such neuroscience-based tools would need to have good construct validity (correspond meaningfully to relevant clinical symptoms), be reproducible (have good test–retest reliability), be easily interpretable by individual clinicians and patients during their treatment decision-making process, and readily incorporable into psychiatry residency and psychology internship education. Finally, although we have focused on several psychiatric disorders in this review to illustrate common and divergent themes, other disorders are worthy of future considideration including posttraumatic stress disorder and autism spectrum disorders.

## Conclusion

In summary, the study of interoception in psychopathology represents a viable avenue for clinical and translational research in psychiatry with (1) a well-established conceptual framework relevant to a variety of psychiatric disorders, (2) a neural basis, (3) measurable biomarkers, (4) interdisciplinary appeal that may help bridge the gap between disciplines, and (5) numerous transdiagnostic points of intervention for understanding and improving mental health outcomes.

## Author Contributions

SK conceived of the work. SK and RL conducted the literature search. SK and RL drafted and revised the work.

## Conflict of Interest Statement

The authors declare that the research was conducted in the absence of any commercial or financial relationships that could be construed as a potential conflict of interest.
