# Supplementary material for: Can Interoception Improve the Pragmatic Search for Biomarkers in Psychiatry?
Source: Front Psychiatry. 2016 Jul 25;7:121. doi: 10.3389/fpsyt.2016.00121 (PMC4958623; doi:10.3389/fpsyt.2016.00121)
Supplement: Supplementary file 1 [file Data_Sheet_1.PDF]

Further definitions of some different facets of interoception.

Interoceptive attention (IAtt): The ability to direct attentional resources towards the source of internal body sensations. IAtt can be captured (i.e., triggered involuntarily) in a stimulus dependent, 'bottom up' manner, or directed purposefully in a 'top down' manner.

Interoceptive Detection (ID): Interoceptive detection is the ability to detect the presence or absence of a stimulus. It is a binary variable, similar to detection of a light source when it is switched on or off, or a judgment about whether a river is flowing or not.

Interoceptive magnitude (IM): Interoceptive magnitude reflects the intensity with which an internal bodily event has occurred. IM is therefore a stimulus parameter reflecting the amount of signal. It is a continuous variable, e.g., reflecting how much dyspnea is present, and is typically gauged from subjective reports from the individual.

Interoceptive Discrimination (IDisc): Interoceptive discrimination is an individual's ability to localize interoceptive sensation within a specific physiological system and to differentiate it from non-interoceptive sensations. It often requires the ability to localize sensations to specific regions within the body (as opposed to elsewhere inside the body), or relative to an external signal. Examples include the ability to distinguish a feeling of fullness after a meal from an irritating cough, or from the noise of a television in the background. IDisc may also require the ability to separate different sensations from within the same interoceptive source, for example, as occurs when swallowing a food bolus (proximal vs. distal esophageal sensation and subsequent gastric deposition).

Interoceptive Accuracy (IAcc): Interoceptive accuracy is the ability to the ability to precisely and correctly monitor changes in internal body state. IAcc is synonymous with interoceptive sensitivity, and it has been the most commonly studied facet. Typical approaches involve the simultaneous measurement of an objective marker (e.g., heart rate, degree of inspiratory breathing load), the subjective experience of the individual (e.g., counted heart rate, detection or intensity of breathing difficulty), and subsequent calculation of the relationship between them. Subjective variables may be dichotomous (e.g., sensation present or absent, as in ID) or continuous (e.g. how intense the stimulus is, as in IM, or IDisc). The interoceptive stimuli that are attended to may be discrete and abrupt (as in the heartbeat) or continuous and prolonged (as in gut or bladder fullness). IAcc necessarily depends upon IAtt given the reliance on attentional mechanisms for generating accuracy estimates. Common examples of IAcc measurements include: 1) d' for heartbeat detection tasks (a signal detection metric (1, 2, 3), cardiac error score for heartbeat counting tasks (4, 5), 2) cross correlations between heart rate and dial tracings of perceived intensity following adrenergic infusion (6, 7), 3) percent accuracy for detection of breathing occlusion (8), 4) intraclass correlation between bladder volumes and urinary urge (9) and 5) cross correlations between respiratory trace and slider tracings of the perceived phase and depth of respiration (10).

Interoceptive Self-report (ISR): Interoceptive self-report is the ability to reflect upon one's autobiographical experiences of interoceptive states, make judgments about their outcomes, and describe them through verbal or motor responses. It is most commonly assessed experimentally using instruments or scales (11, 12, 13, 14, 15) but it may also be assessed by asking questions about personal observations related to task performance. For example, in a study in which experienced meditators did not have different I_{Acc} on a heartbeat detection task, they exhibited differences in I_{SR}, reporting that the task was easier and their performance was improved relative to nonmeditators (16). In another study, performance on a heartbeat counting task was positively correlated with self-reported confidence estimates only in individuals with high but not low interoceptive accuracy scores (Garfinkel et al., 2015). There are likely to be many other constructs that can be identified within this level. For example, it may be possible to investigate dissociations between an individual's self-report of their interoceptive experiences versus their partner report (e.g., someone who knows the participant well and has had regular opportunities to interact with and observe them in a variety of situations, an approach that has been utilized for neurological samples (17)). Accordingly, the self-report component may be viewed as one of the most complex facets of IA.

REFERENCES:

1. Brener J, Liu XQ, Ring C. A method of constant stimuli for examining heartbeat detection – comparison with the Brener-Kluvitse and Whitehead methods. Psychophysiology. 1993;30(6):657-65.
2. Khalsa SS, Rudrauf D, Tranel D. Interoceptive awareness declines with age. Psychophysiology. 2009;46(6):1130-6.
3. Jones GE, Oleary R, Pipkin B. A comparison of Brener-Jones and Whitehead procedures for assessing cardiac awareness. Psychophysiology. 1982;19(5):567-.
4. Herbert BM, Ulbrich P, Schandry R. Interoceptive sensitivity and physical effort: implications for the self-control of physical load in everyday life. Psychophysiology. 2007;44(2):194-202.
5. Koch A, Pollatos O. Cardiac sensitivity in children: sex differences and its relationship to parameters of emotional processing. Psychophysiology. 2014;51(9):932-41.
6. Khalsa SS, Craske MG, Li W, Vangala S, Strober M, Feusner JD. Altered interoceptive awareness in anorexia nervosa: Effects of meal anticipation, consumption and bodily arousal. The International journal of eating disorders. 2015;48(7):889-97.
7. Khalsa SS, Rudrauf D, Feinstein JS, Tranel D. The pathways of interoceptive awareness. Nat Neurosci. 2009;12(12):1494-6.
8. Zhao W, Martin AD, Davenport PW. Magnitude estimation of inspiratory resistive loads by double-lung transplant recipients. 1985). 2003;94(2):576-82. Epub 2002 Oct 11.
9. Heeringa R, van Koeveringe GA, Winkens B, van Kerrebroeck PE, de Wachter SG. Degree of urge, perception of bladder fullness and bladder volume--how are they related? J Urol. 2011;186(4):1352-7. doi: 10.016/j.juro.2011.05.050.
10. Daubenmier J, Sze J, Kerr CE, Kemeny ME, Mehling W. Follow your breath: respiratory interoceptive accuracy in experienced meditators. Psychophysiology. 2013;50(8):777-89.
11. Labus JS, Mayer EA, Chang L, Bolus R, Naliboff BD. The central role of gastrointestinal-specific anxiety in irritable bowel syndrome: further validation of the visceral sensitivity index. Psychosom Med. 2007;69(1):89-98.
12. Shields S, Mallory ME, Simon A. The body awareness questionnaire: reliability and validity. J Pers Assess. 1989;53(4):802-15.
13. Porges S. Body perception questionnaire: University of Maryland; 1993.
14. Mehling WE, Price C, Daubenmier JJ, Acree M, Bartmess E, Stewart A. The Multidimensional Assessment of Interoceptive Awareness (MAIA). PLoS One. 2012;7(11):e48230.
15. Garfinkel SN, Seth AK, Barrett AB, Suzuki K, Critchley HD. Knowing your own heart: distinguishing interoceptive accuracy from interoceptive awareness. Biol Psychol. 2015;104:65-74.
16. Khalsa SS, Rudrauf D, Damasio AR, Davidson RJ, Lutz A, Tranel D. Interoceptive awareness in experienced meditators. Psychophysiology. 2008;45(4):671-77.
17. Barrash J, Asp E, Markon K, Manzel K, Anderson SW, Tranel D. Dimensions of personality disturbance after focal brain damage: investigation with the Iowa Scales of Personality Change. J Clin Exp Neuropsychol. 2011;33(8):833-52.
