# Supplementary material for: Can Interoception Improve the Pragmatic Search for Biomarkers in Psychiatry?
Source: Front Psychiatry. 2016 Jul 25;7:121. doi: 10.3389/fpsyt.2016.00121 (PMC4958623; doi:10.3389/fpsyt.2016.00121)
Supplement: Supplementary file 2 [file Data_Sheet_2.PDF]

Comprehensive list of search terms used to identify publications related to interoception (box inset) and some of the facets of interoception (remaining terms).

Interocept*	Visceral +	Cardiac +	Heartbeat +	Palpitation +	Respiratory +
Interocept*+	Sens*	Sens*	Sens*	Sens*	Sens*
Awar*	Awar*	Awar*	Awar*	Awar*	Awar*
Sens*	Hypersen*	Percept*	Percept*	Percept*	Percept*
	Stim*	Discrim*	Discrim*	Discrim*	Discrim*
		Detect*	Detect*	Detect*	Detect*
		Hypers*	Hypers*	Hypers*	Hypers*
		Hypos*	Hypos*	Hypos*	Hypos*
Dyspnea+	Esophageal +	Oesophageal +	Stomach +	Gastric +	Colon +
Sens*	Sens*	Sens*	Sens*	Sens*	Sens*
Awar*	Stim*	Stim*	Stim*	Stim*	Stim*
Percept*	Percept*	Percept*	Percept*	Percept*	Percept*
Discrim*	Discrim*	Discrim*	Discrim*	Discrim*	Discrim*
Detect*	Disten*	Disten*	Disten*	Disten*	Disten*
Hypers*	Hypers*	Hypers*	Hypers*	Hypers*	Hypers*
Hypos*	Hypos*	Hypos*	Hypos*	Hypos*	Hypos*
Rectal +	Colorectal +	Anorectal +	Bladder +	Hypoglycemia +	Hyperglycemia +
Sens*	Sens*	Sens*	Sens*	Awar*	Awar*
Stim*	Stim*	Stim*	Stim*	Percept*	Percept*
Percept*	Percept*	Percept*	Percept*	Discrim*	Discrim*
Discrim*	Discrim*	Discrim*	Discrim*	Hypers*	Hypers*
Disten*	Disten*	Disten*	Disten*	Hypos*	Hypos*
Hypers*	Hypers*	Hypers*	Hypers*		
Hypos*	Hypos*	Hypos*	Hypos*		
Panic and Sens* +	Panic and Sens* +				
Lactate	Yohimbine				
Isoproterenol	Epinephrine				
Carbon-Dioxide	Doxapram				
